# Healthcare professionals’ experiences of telehealth adoption in palliative care: A cross-sectional survey

**DOI:** 10.1177/26323524261437366

**Published:** 2026-07-23

**Authors:** Ann Kirby, Donal Griffin, Ciara Heavin, Fiona Kiely, Frances J. Drummond, Ciara McGrath

**Affiliations:** 1 Department of Economics, Cork University Business School, University College Cork, Ireland; 2 Institute of Applied Health Research, University of Birmingham, UK; 3 Department of Business Information Systems, Cork University Business School, University College Cork, Ireland; 4 Marymount University Hospital & Hospice, Cork, Ireland; 5 Breakthrough Cancer Research, Cork, Ireland

**Keywords:** telehealth, palliative care, healthcare professionals (HCPs), Unified Theory of Acceptance and Use of Technology (UTAUT) model

## Abstract

**Background:**

Research has revealed a dramatic rise in the adoption of telehealth by healthcare professionals (HCPs). Limited evidence exists focusing solely on HCPs’ adoption and use of telehealth in palliative care and lacks nuance about when and how telehealth is clinically appropriate.

**Objective:**

The aim is to deepen understanding of HCPs’ behavioural intention (BI) regarding the adoption and use of telehealth in palliative care, and to examine their current patterns of telehealth use.

**Methods:**

Cross-sectional data were collected from HCPs working in palliative care between July 27th and September 22nd, 2023. A probit analysis examined the difference between users and non-users of telehealth in palliative care, while a multiple linear regression assessed the association between a HCPs’ BI of using telehealth and Performance Expectancy, Effort Expectancy, Facilitating Conditions, and Social Influence.

**Results:**

Perceived benefits to clinical performance were associated with increased willingness to adopt telehealth. Workplace culture, experience, opportunity to use telehealth, and peer encouragement were highlighted as important contributors to a supportive environment. However, despite positive BIs, many previous users reported infrequent use in practice, suggesting a gap between intention and routine clinical integration.

**Conclusion:**

HCPs recognised the benefits of telehealth, but concerns remained regarding treating and managing physical symptoms in palliative care. Telephone-based services were still primarily used, though hybrid models were recognised as needed at times in some clinical situations. Patterns of use suggested distinct user groups, indicating that telehealth implementation requires tailored training, organisational supports, and clear escalation pathways for transitioning from virtual to in-person care. Addressing these factors may support more sustainable and clinically appropriate telehealth delivery in palliative care, with potential benefits for patient outcomes and experiences.

## Introduction

Palliative care is a specialised form of medical care that focuses on the unique needs of individual patients and families as they cope with the effects of progressive serious illness and associated treatments. In palliative care, a multidisciplinary team of healthcare professionals (HCPs) works together to ease pain, reduce distress, and support patients’ overall well-being. Simultaneously, the needs of family members are identified and addressed in a concerted effort to reduce bereavement morbidity. During the palliative phase of their disease, many people are not hospital-based, with their disease managed sufficiently to enable them to continue to live at home or in a community setting. In fact, research has shown that many spend their remaining time at home.^1–3^ These services are essential to meet the needs of patients facing serious, often terminal conditions, aiming to improve and support both patients and their families. Palliative care emphasises holistic well-being and individualised care planning that aligns with a patient’s values and preferences. However, of the 56.8 million people globally in need of palliative care, only 14% can access it.^4^ Access to palliative care needs to improve further and be available in settings outside hospitals – in medical offices and clinics, in post-acute and long-term care facilities, and in patient homes. Therefore, increasing the availability of palliative care services should be a priority for health policymakers globally.

With the introduction of telehealth comes a novel opportunity to leverage technology to extend access to palliative care services. Telehealth is a term often used interchangeably with telemedicine. Telehealth is defined as “the use of medical information that is exchanged from one site to another through electronic communication to improve a patient’s health.”^5^ Indeed, more broadly, telehealth technologies, tools, and services are becoming a vital component of the healthcare system.^6^ Telehealth offers online activities and resources designed to educate both HCPs, patients, and communities^7–9^ and its acceptance has increased among patients across OECD countries since COVID-19,^10,11^ with telehealth supporting people with advanced illness – and their families or carers – through remote symptom monitoring and management.^12,13^

Although the COVID-19 health emergency has passed, telehealth in palliative care will remain, in part, because of increased global care needs and technology advances. Clinical trials show that timely access to specialist palliative care improves outcomes for patients and their families compared with usual care.^14–16^ Globally, there is also recognition of palliative care patients’ preference to remain at home, thereby relieving pressure in overcrowded hospitals.^17^ While access barriers remain, particularly for socially disadvantaged groups^18^ and the elderly,^19^ research reveals that a more flexible hybrid approach combining in-person and telehealth visits is needed to support patient-provider connections and trust.^20^ As demand for palliative care grows, investment in digital health is a priority to relieve the pressure of delivering services.^21^ In Ireland, the National Broadband Plan aims to improve internet access and deliver high-speed broadband services, helping reduce the digital divide between urban and rural areas and strengthening the effectiveness of telehealth.^22^ Telehealth is an alternative solution that improves access to HCPs^23^ at a lower relative cost.^24^ In the context of sustainability, metrics for digital health interventions suggest significant cost savings and increased efficiencies, which in turn contribute to the UN sustainability goals.^25^

COVID-19 placed a disproportionate impact on palliative care services, placing strain on the workforce.^26^ Despite success with telehealth in palliative care, some HCPs remain hesitant, citing concerns about the overall quality of care (e.g. lack of physical examination), liability potential, lack of training, and hardware expenses, which are still prevalent.^27,28^ While not all types of care can be replaced with online consultations,^27^ early evidence suggests it can bolster access during disruptions to essential services,^29^ and its adoption continues to accelerate.^30^ Research emphasises the need to evaluate HCPs’ perceptions of digital technologies to inform future training and multi-stakeholder incentives,^31^ which is crucial within palliative care, given the complexities of providing care to patients with serious illness. A clearer understanding of how digital tools can be effectively integrated to further enhance communication, decision-making, and overall care delivery is needed. Given the limited evidence focused solely on HCPs’ use of telehealth in palliative care, examining acceptance and intention to use telehealth among HCPs is essential to further advance understanding in this setting.

This study draws on the Unified Theory of Acceptance and Use of Technology (UTAUT) model as a lens to determine the relationship between intention to use telehealth in palliative care and the predictors therein.^32^ The UTAUT is a widely used Information System (IS) theory that provides valuable insights to explain and predict adoption and use of technology. It is expected that some HCPs already use telehealth in palliative care, while others do not. Understanding the differences between users and non-users, and describing how telehealth is currently being used, may provide additional insight into its role in palliative care. An improved understanding of HCPs’ adoption and use of telehealth is crucial for planning the future delivery of palliative care services. The overall aim is therefore to advance understanding of the factors that influence HCPs’ behavioural intention (BI) to adopt and use telehealth, while also examining actual patterns of use.

## Theoretical model

**Figure 1.**
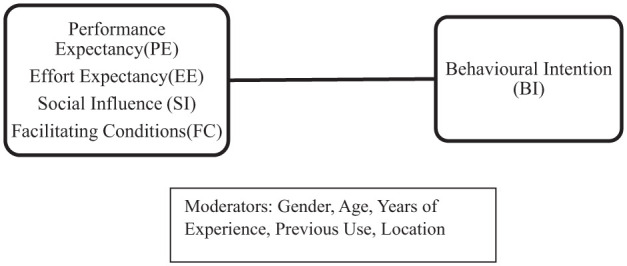
Unified Theory of Acceptance and Use of Technology (UTAUT) model. Source: Adapted from Venkatesh et al.^32^

User acceptance of new IS/IT is widely considered in contemporary research and has resulted in various theoretical models for explaining individuals’ intention to use.^33^ Drawn from the fields of IS, Sociology, and Psychology, the Technology Acceptance Model (TAM), which measures willingness and acceptance of use of technology,^34^ and the theory of reasoned action,^35,36^ posit that individual behaviour is driven by personal BIs.^36^ These theories offer diverse explanations of IS/IT acceptance and usage, considering factors such as technology attributes and contextual factors. Building on this foundation, we rely on the UTAUT model, which is derived from these theories. BI is a measure of the strength of a HCPs’ intention to use technology,^37^ in this case, telehealth in palliative care. This is determined by four main constructs: Performance Expectancy (PE), Effort Expectancy (EE), Social Influence (SI), and Facilitating Conditions (FC; See Figure 1).

PE is the degree to which an individual believes that using a new system will help them to improve performance in their job.^32^ PE measures HCPs’ perceived usefulness of the technology to perform certain activities. Several studies have shown that PE is a determinant of the BI to use telehealth by HCPs.^28,38^

Therefore, we propose the following hypothesis:

**Hypothesis 1:** PE positively influences the BIs of HCPs in palliative care to use telehealth.

EE is the degree of ease associated with the consumer’s use of a technology.^39^ EE can have a significant influence on BI^40^; when HCPs perceive a technology as easy to use, then they are more likely to adopt it. Therefore, we hypothesise:

**Hypothesis 2:** EE positively influences the BIs of HCPs in palliative care to use telehealth.

SI is defined as the degree to which an individual perceives that others who are important to them believe he or she should use the new system.^32^ SI is similar to the concept of subjective norms, where the behaviour of people is adjusted to the perception of others about them. The effect of SI is significant when the use of technology is mandated.^32^ In this context, individuals might use technology due to compliance requirements, but not because of personal preferences.^41^ Therefore, we hypothesise that:

**Hypothesis 3:** SI positively influences the BIs of HCPs in palliative care to use telehealth.

FCs are the degree to which an individual believes that an organisational and technical infrastructure exists to support the use of the new technology.^32^ FCs is the influence of available infrastructure on the intention to use technology by the HCP and indicates that users require system availability at their place of work.^32^ Existing research suggests that the absence of technological infrastructure could demotivate users to adopt a new technology.^42^ Using the UTAUT model, a relationship between users and FC was found,^38,39^ providing a structured way to assess why healthcare providers may accept or resist telehealth solutions.

**Hypothesis 4:** FCs positively influence the BIs of HCPs in palliative care to use telehealth.

The exogenous latent variables (PE, EE, SI, FC) are not directly observed but inferred from multiple observed (i.e. measured) indicators (see Table 1). The endogenous latent variable BI cannot be measured directly but is affected by these variables. The exogenous latent variables (PE, EE, SI, FC) are related to the endogenous latent variable (BI), which is associated with the use of technology. Moderator variables can include, for example, age, gender, years of experience, and voluntariness of use (i.e. use of technology can be voluntary or mandatory) based on previous research,^28,43^ which affect the nature of the relationship between the indicators and constructs. Voluntariness of use was not included, as use of technology is voluntary in this context. For this research, the location of the HCP specialised in palliative care was included, as some treat only inpatients predominantly and therefore the necessity to use telehealth is lower. The role of the HCP and type of contract were also included, as the type of contract (e.g. management) and some roles may have greater opportunity to use telehealth compared to others. Research has found a rise in the adoption of telehealth by HCPs,^11^ therefore there is an expectation that some HCPs are likely to be previous users of telehealth in palliative care. This is also controlled for in the model.

**Table 1.** Description of the constructs.

Construct	Description	No. of Q.
Intention to use (BI1–BI4)	The questions measured their current expectations and intentions around use of telehealth	4
Performance expectancy (PE1–8)	The questions measured their opinion on the usefulness of telehealth	8
Effort expectancy (EE1–3)	The questions measured their opinion about ease of use of telehealth	3
Social influence (SI1–3)	The questions aimed to assess the culture and attitudes they perceive in their current workplace in relation to telehealth	3
Facilitating conditions (FC1–4)	The questions aimed to assess their opinion about organisational characteristics that facilitate telehealth	4

Source: The questions were adapted from Rouidi et al.^43^ and Venkatesh et al.^32,41^ (See Appendix A).

PE: performance expectancy; EE: effort expectancy; SI: social influence; FC: facilitating conditions; BI: behavioural intention.

## Methods

The research methodology applies a probit regression to determine whether there is a significant difference between users and non-users of technology and a multiple regression to examine the association between the predictors PE, EE, SI, and FC and the BIs of HCPs when using telehealth in palliative care.

### Data collection

A dedicated, cross-sectional, anonymous online survey (using Qualtrics) was designed, piloted, and disseminated to consultants, nurses, dieticians, speech and language therapists, pastoral care workers, physiotherapists, medical social workers, pharmacists, and healthcare assistants working in palliative care in Ireland and Northern Ireland between July 27th and September 22nd, 2023. This research targeted HCPs working in palliative care. A sample of the target population assisted in face validity on a voluntary basis. The title and purpose of the survey described the intended target audience (i.e. HCPs working in specialist palliative care).

There was no current complete list of HCPs working in palliative care available as a sample frame. Therefore, the sample frame included members of the Irish Association for Palliative Care (*n* = 280), Irish Palliative Medicine Consultants Association (*n* = 61), and the All-Ireland Institute of Hospice and Palliative Care (AIIHPC; *n* = 607). All members were sent an introductory email along with the survey and a subsequent reminder email. As HCPs can choose to be a member of all three organisations, there may be duplication, which inflates the population size (*N* = 948) and affects the accuracy of the calculated response rate. However, it could be argued that the AIIHPC is a more representative measure of the population. These were the only recruitment channels used. Given the accelerated use of telehealth since COVID-19, it was expected that some HCPs would be previous users of telehealth services while others would remain non-users. Therefore, there was the potential for overrepresentation of individuals with positive telehealth experiences and limited capture of non-users. Consequently, additional questions were included in the survey to establish a better understanding of the actual use of telehealth.

HCPs were eligible if they were >18 years old, had the ability to read English, and worked in specialist palliative care. The survey consisted of questions divided into three parts: (1) demographic characteristics, (2) previous experience of telehealth, (3) constructs around intention to use telehealth within palliative care based on the UTAUT model. These constructs were standardised on a Likert scale (where 1 = strongly disagree and 5 = strongly agree). The constructs measured an individual HCPs BI to use telehealth technologies (BI) based on the predictors PE, EE, FC, and SI. The Likert scale measuring individual questions for each construct was adapted from Rouidi et al.^43^ and Venkatesh et al.^32,41^ to measure the intention to use telehealth in palliative care, and a description of these constructs is included in Table 1.

The survey characterised telephone consultations, video-enabled care, remote patient monitoring, or other forms of telehealth as mutually inclusive options to measure the use of telehealth. Within the pilot, it was revealed that most participants were familiar with these options. Video-enabled care was defined as a web-based solution that helps healthcare providers offer video call access to their services while remote monitoring involves the use of digital technologies to acquire patient data outside of the traditional clinical environment.^44^ The nature of the telehealth service provided (i.e. symptom management, medication review, care planning, etc.) was not examined.

### Statistical analysis

Descriptive statistics were provided for the full sample of respondents, including users and non-users of telehealth in palliative care. Mean scores were constructed as aggregates of the individual items BI, PE, SI, EE, and FC, which ranged from a score of 1–5 (see Table A1). A high score indicated a high intention to use telehealth technology (4–5), and a low score (1–2) was the opposite. A score of 3 indicated a neutral or inconclusive score. The mean scores for each of the constructs were aggregated and examined, and differences in mean scores for users and non-users were determined using the Mann-Whitney *U* (if the mean construct was not normally distributed) and independent *t* tests (*p* < 0.05). Little’s (1998) test examined whether there were significant differences between the means of different missing value patterns to ensure that the missing values were random and did not create bias if excluded.^45^ Then, Cronbach’s alpha was calculated to check for the internal consistency of the constructs and ensure the reliability of the measurement.

To determine whether the characteristics of users and non-users were significantly different, a probit model was employed, and marginal effects at the mean were estimated.

$$\begin{array}{l} P(Y=1|X)=\phi (\beta_{0}+\beta_{1}\left(BI\right)+\beta_{2}\left(PE\right)+\beta_{3}\left(EE\right)\\ \;\;\;\;\;\;\;\;\;\;\;\;\;\;\;\;\;\;\;\;+\beta_{4}\left(SI\right)\;+\beta_{5}\left(FC\right)+\beta_{6}(\mathrm{MV}))+\varepsilon \end{array}$$

The dependent variable *Y* was binary (i.e. 0 = non-user, 1 = user). *φ* transforms the linear combination of the predictors BI, PE, EE, SI, and FC into a probability that lies between 0 and 1, controlling for moderating variables (MVs; e.g. an aggregate of gender, age, years of experience, location, role, and type of contract). Role and type of contract were insignificant in the final probit and were therefore excluded.

A multiple linear regression was applied, given the nature of the data (i.e. continuous and categorical), the cross-sectional design, and the relatively small sample size. In the context of TAMs (e.g. UTAUT), a multiple regression has previously been used to measure the impact of key factors on intention to use telehealth.^46^

$$\begin{array}{l} \mathrm{BI}=\beta_{0}+\beta_{1}\left(PE\right)+\beta_{2}\left(EE\right)+\beta_{3}\left(SI\right)\\ \;\;\;\;\;\;\;+\beta_{4}\left(FC\right)+\beta_{5}(\mathrm{MV})+\varepsilon \end{array}$$

The multiple regression model examined the relationship between BI to use telehealth in palliative care and the predictors PE, EE, SI, and FC, controlling for MVs (e.g. an aggregate of gender, age, years of experience, previous experience, and location of the HCP). Assumptions of multiple regression were assessed, including normality of the residuals, linearity, homoscedasticity, and multicollinearity. The data were analysed using Stata version 18.^47^

### Ethical considerations

Ethical approval was received from University College Cork’s Social Research Ethics Committee Log 2023-089 April 2023. Participation in the survey was voluntary, and informed consent was obtained. Only anonymous data were collected.

## Results

### Descriptive statistics

The sample comprised 73 HCP respondents. The response rate was approximately 13% (assuming a *N* = 948). As individuals may have belonged to multiple lists, the effective denominator is uncertain, making the response rate an approximation. Within the sample, there was a mix of previous users (*n* = 45) and non-users of telehealth in palliative care (*n* = 28). Due to item non-response, the sample size was reduced to 59 respondents (i.e. 37 previous users and 22 non-users of telehealth in palliative care).

Among the HCPs who responded (*n* = 59), the majority were female (83%), with a median age of between 45 and 54 years (42%), and most had 11 or more years of professional experience (64%). Respondents were either doctors (31%), nurses (44%), or other health and social care practitioners (25%; e.g. dieticians, physiotherapists, speech therapy, medical social workers, pharmacist, pastoral care workers, occupational therapists). HCPs worked in hospices (32%), hospitals (10%), or community settings (29%), with the remaining respondents located in two or more settings (27%) within the Irish healthcare system (hospice, hospital, or community). Forty-three percent of respondents had a management role and 63% were working full-time (see Table A2).

The survey differentiated between the characteristics of HCP users (*n* = 37 (63%)) and non-users of telehealth in palliative care (*n* = 22 (37%); see Table A2). The distribution of contract type and gender was broadly similar between groups; however, differences were observed across age, experience, role, location, and management status. Within telehealth users (*n* = 37), the majority were aged 45–54 years (49%), had 11 or more years of experience (67%), were mostly nurses (48%), and worked in community settings (43%). The proportion of users in a management role or a non-management role was equal (45%). Within non-users of telehealth (*n* = 22), the majority were older, aged 55 years or more (41%), had 11 or more years of experience (58%), and were nurses (36%). They mostly worked in a hospice (50%) and were not in management roles (50%).

### Profile of telehealth use in palliative care

Within the sample, 37 HCPs reported previous use of telehealth in palliative care. Of those, 65% of HCPs (*n* = 24) reported using telehealth within the previous 6 months. A further 8% of HCPs (*n* = 3) reported using telehealth in palliative care within the previous 7–12 months. While 27% of HCPs (*n* = 10) reported not using telehealth in more than 1 year. Respondents were also asked to reflect on their experiences of using telehealth in palliative care. In a typical week, 41% (*n* = 15) reported “never” or “rarely” using telehealth in palliative care, while the remaining 59% (*n* = 22) used telehealth in palliative care “sometimes,” “often,” or “always” during the week.

Regarding duration of use, 14% (*n* = 5) used telehealth for less than an hour per week, 22% (*n* = 8) for 1–3 h, 19% (*n* = 7) for 4–6 h, and 17% (*n* = 6) for 7 or more hours. On average, HCPs used telehealth in palliative care about 1–3 h (mean = 1.83) per week (see Table 2). Although 28% (*n* = 10) reported spending zero hours per week using telehealth, this was inconsistent with the proportion reporting “never” using telehealth weekly (17%; *n* = 6; see Table 2). This discrepancy may reflect recall limitations.

**Table 2.** Profile of telehealth use in palliative care.

Variable	Frequency (%), *n* = 37
Pre-COVID use of telehealth	
Yes	9 (24%)
No	28 (76%)
Most recent use of telehealth	
Within the past 0–6 months	24 (65%)
Within the past 7–12 months	3 (8%)
More than 12 months	10 (27%)
Weekly use	
Never	6 (17%)
Rarely	9 (24%)
Sometimes	9 (24%)
Often	10 (27%)
Always	3 (8%)
Hours per week^a^	
0	10 (28%)
<1	5 (14%)
1–3	8 (22%)
4–6	7 (19%)
7+	6 (17%)
Overall mean	1.83 h (SD = 1.46 h)
Types of telehealth	
TC	18 (49%)
VEC	1 (3%)
RHM	0 (0%)
TC and VEC	15 (40%)
TC and RHM	1 (3%)
TC, VEC, and RHM	2 (5%)

SD: standard deviation; TC: telephone consultation; VEC: video-enabled care; RHM: remote health monitoring.

a Hours per week *n* = 36 due to item-level missingness.

Further inspection of the duration and frequency variables revealed broadly similar patterns of use, except for a small number of respondents who selected “rarely” for frequency of weekly use while reporting zero hours of telehealth use per week (*n* = 4). These respondents remained classified in the “rarely” user category, although based on reported hours, they could alternatively be considered as “never” users. This minor mismatch may be due to recall differences, and as both categories represent minimal or no telehealth use, a reclassification would not meaningfully alter the overall findings.

Regarding modality, 49% (*n* = 18) used the telephone as their main mode of communication. Forty-three percent (*n* = 16) used either telephone consultations and video-enabled care (40%) or video-enabled care alone (3%). Only 3% (*n* = 1) used the telephone and remote health monitoring, while 5% (*n* = 2) reported using all three telehealth modalities.

### Intention to use telehealth in palliative care and the predictors

Generally, there was a positive response (i.e. (strongly) agree) to BI to use telehealth, with 54% of previous users and non-users reporting that they would like to increase their use and 63% reporting an expectation of use by their organisation (see Table A1). However, only 27% indicated that they anticipated using telehealth in the next 12 months. The highest response rate was associated with a perceived reduction of use in telehealth, with 52% “(strongly) disagreeing” with this statement. This suggests that HCPs have an intention to use telehealth in palliative care where appropriate. Missing values were found for the BI outcome variables (BI1–4). Little’s (1998) test showed that these values were missing at random (*p* = 0.88; see Table A3). (Note the construct BI4 had an inverse interpretation compared to the other BI constructs (BI1–4); therefore, the mean construct was recoded for a more explicit interpretation.) See Table A1.

There was a positive response to the measurement of PE, with more than 60% of respondents across both users and non-users of telehealth “(strongly) agreeing” that telehealth is useful (71%), convenient (73%), improves integration of patient care (64%), improves access (68%), supports the effective allocation of staff resources (66%), and generates cost savings (75%). While 59% “(strongly) agreed” that telehealth can support the delivery of quality palliative care, less than 28% believed that patients’ physical symptoms can be adequately addressed using telehealth (see Table A1).

The construct EE shows that more than 50% of both users and non-users “(strongly) agree” that using telehealth is simple (59%) and easy to use (73%). However, less than 31% “(strongly) agree” that they rarely experience issues or problems using telehealth technology. For the construct FC, more than 60% “(strongly) agree” that they have access to adequate ICT support (64%) and a reliable internet connection (73%). However, 51% “(strongly) disagreed” when asked if they had received adequate education and training. For SI, only 34% “(strongly) agreed” that they were encouraged by managers to use telehealth, 44% were aware of the routine use of telehealth by colleagues, and 39% were aware of colleagues who were enthusiastic about the use of telehealth and championed its use in the organisation. Using *t*-tests and the Mann-Whitney *U* test, there was no significant difference found between the mean scores of the constructs for users and non-users of telehealth in palliative care (see Table A3).

### Probit and multiple regression results

Overall, Cronbach’s alpha test for reliability ranged from 0.642 to 0.915 (see Table A4). EE showed a somewhat lower internal consistency (Cronbach’s alpha = 0.66). Subsets of items (EE1–3) were included and excluded from the construct; however, the alpha value remained just slightly below 0.70. The construct was included, given that generally, reliability numbers greater than 0.6 are considered acceptable in technology acceptance literature.^48^ This result might be influenced by the relatively higher percentage in the neutral section (ranging from 20% to 42%), suggesting a lack of established attitudes towards ease of use.

A probit determined whether the characteristics of users and non-users were significantly different. User/non-user status was specified as the dependent variable, with the predictors including age, gender, experience, and location, as well as the constructs BI, EE, PE, FC, and SI. Multicollinearity was found between age and years of experience; therefore, only experience was included in the model. Gender was excluded due to the disproportionate representation of female respondents (83%). Marginal effects at the mean were estimated and are presented in Table 3.

**Table 3.** Probit of users and non-users of telehealth technology in palliative care.

Variables	d*y*/d*x*	SE
Experience		
6 years+	0.31^a^	0.16
Location		
>1 location	0.33^b^	0.15
Constructs		
BI	0.05	0.10
EE	−0.12	0.12
PE	0.03	0.11
FC	0.01	0.12
SI	0.18^b^	0.09

PE: performance expectancy; EE: effort expectancy; SI: social influence; FC: facilitating conditions; BI: behavioural intention.

d*y*/d*x* reflects the average marginal effects: 1–5 years experience and based in one location are the base categories. The constructs are continuous variables.

a Value is significant at *p* < 0.01,

b Values are significant at *p* < 0.05.

Value is significant at *p* < 0.10, *n* = 59 (i.e. users = 37 (1), non-users = 22 (0)).

Relative to those with 15 years’ experience, more experienced HCPs (*p* = 0.055) were more likely to be users of telehealth in palliative care. HCPs working in more than one location (hospice, hospital, or community) were more likely to be users (*p* = 0.030) compared to HCPs located in one setting. Moreover, a 1 unit increase in the mean level of agreement for SI was associated with a 18% higher predicted probability of being a user (*p* = 0.055).

Given the empirical results of the probit, the multiple linear regression was adjusted to include previous experience (i.e. whether you were a user or non-user), location, and years of experience in palliative care. Because of the theoretical considerations of the model, all the variables were included rather than using a stepwise regression. Several assumptions were examined, including multicollinearity (using the variance inflation factor (VIF) diagnostic), normality of the residuals (Shapiro-Wilks test), linearity, and heteroscedasticity. There was some correlation among the predictor variables, but only moderate to low levels, indicating the absence of multicollinearity problems (see VIF, Table 4). Normality of the residuals was significantly skewed, as BI was skewed to the left with a range of 1.5–5. This was expected as the descriptive statistics found that most BIs were either “neutral” or above in terms of agreement (see Table A1). After examining the residuals, linearity was found between the outcome variable and the predictors, while robust standard errors were employed to address heteroscedasticity (Note: Transforming BI due to skewness had no impact on significance and the relative importance of the predictors; therefore, BI remained the same). In summary, there was no evidence of problematic multicollinearity, residuals were skewed due to high BI scores, but transformations did not change significance, and robust standard errors were reported.

**Table 4.** A multiple regression of behavioural intention to use telehealth in palliative care.

Variables	Coeff.	SE	VIF
EE	0.07	0.13	1.33
PE	0.39^a^	0.13	1.34
FC	−0.03	0.12	1.73
SI	0.10	0.12	1.40
>1 location	0.16	0.24	1.26
6 years+	0.03	0.19	1.10
Previous user	0.10	0.20	1.26
Constant	1.39^a^	0.50	

PE: performance expectancy; EE: effort expectancy; SI: social influence; FC: facilitating conditions.

a Value is significant at *p* < 0.01, adjusted *R*^2^ is 22%, *n* = 59, *F* = 3.63, *p* = 0.003. One to five years experience and based in one location are the base categories.

On estimation of the model, only PE was significantly associated with HCPs’ intention to use telehealth in palliative care (*p* = 0.004; see Table 4). Within the construct PE (1–8Q), more than 70% “(strongly) agreed” with the benefits of telehealth in terms of cost savings, usefulness, and level of convenience (see Table A1). The level of agreement for patient access (68%), effective allocation of resources (66%), improvement in integrative care (64%), and quality of care (59%) was slightly lower. Just over one in four (25%) respondents believed that a patient'’ physical symptoms could be adequately addressed using telehealth. The constant was also significant (*p* = 0.007), suggesting that, on average, there is a baseline level of use that may be capturing inherent factors such as availability.

## Discussion

From the regression analysis, PE was the only variable significantly associated with HCPs’ intention to use telehealth in palliative care, thus aligning with findings from other healthcare settings.^49,50^ This suggests that when HCPs perceive telehealth as beneficial, their intention to use it is likely higher. Examination of the underlying levels of agreement (i.e. PE1–PE8) showed that most HCPs agreed that telehealth was useful, convenient, and cost-effective. However, only one in four respondents believed that a patient’s physical symptoms could be adequately addressed using telehealth. This gap highlights a practical limitation of telehealth in palliative care and may result in more frequent in-person follow-ups. Consequently, the perceived convenience and cost-effectiveness of telehealth in palliative care may be reduced if virtual consultations need to be supported by face-to-face visits. These findings highlight the importance of creating more flexible, responsive care pathways that enable patients to transition from virtual to in-person care as needed. However, given the small and potentially self-selecting sample, which includes a high proportion of current telehealth users, these findings should be interpreted with caution.

The probit model examining differences between users and non-users found that SI was significantly associated with the likelihood of being a current telehealth user in palliative care. Instead of simply reflecting peer use, SI was included to capture the culture and attitudes of the workplace, including managerial encouragement and the presence of visible telehealth champions. Examination of the underlying levels of agreement within SI (i.e. S1–S3) revealed that users reported a higher level of agreement across these indicators, suggesting that users are operating in supportive social networks that promote telehealth adoption, which in turn encourages utilisation. Strengthening these organisational supports, through leadership engagement, peer mentoring, and highlighting positive experiences, may therefore support sustainable adoption and acceptance.

FCs assessed HCPs’ perceptions of organisational characteristics that facilitate telehealth use (e.g. availability of telehealth). Although FCs were not significant in the regression results, HCPs working across multiple sites were more likely to use telehealth in palliative care. A possible explanation is that an HCP practising in different settings may treat a diverse patient population with different clinical conditions or care needs, thereby necessitating telehealth to access, maintain, and provide care to patients across locations. In contrast, if the HCP is based full-time in a single setting, they may likely be treating inpatients predominantly, and therefore, the necessity to use telehealth is lower. Another possible explanation is that this may reflect clinical governance and associated medical, legal, and contractual obligations of the HCP, particularly if patients move across different healthcare settings. All explanations are plausible but currently speculative, given the lack of supporting data.

HCPs’ years of experience were positively associated with telehealth use in the probit analysis. This result may be reflecting greater leadership, confidence, and willingness to adopt new practices among more experienced members of staff. Delivering clinical treatment using telehealth can be challenging, particularly when interpreting social cues or managing complex symptoms in an online environment.^28^ Prior experience of using telehealth in routine practice is likely to encourage confidence, thereby supporting continued use.^28^

Overall, HCPs revealed a relatively high level of intention to use telehealth in palliative care. Moreover, the percentage intending to use telehealth in palliative care in the next 6 months is higher than that of those who used it in the previous 6 months. This suggests a positive trend towards the future use of telehealth in palliative care. In contrast, it should be noted that 41% of previous users (*n* = 15) revealed that they “rarely” or “never” used telehealth on a weekly basis. This may indicate lingering resistance to change in terms of technology adoption, or a lack of opportunity to use telehealth because of patient or setting needs. Other explanations for this result could also be related to technical and usability issues, clinical appropriateness, patient mix, or workflow constraints.^25,28,31,51^

### Implications of the results

Understanding existing telehealth use patterns can help identify factors influencing HCP adoption and guide strategies to improve access to care. Infrastructure, technical limitations, psychological factors, and workload concerns remain key barriers.^31^ Training and ongoing support are required across user groups, particularly in conducting remote consultations, including skills for online communication, symptom assessment, and managing complex conversations.

From a clinical perspective, sustained telehealth use in complex care is consistently associated with multicomponent implementation approaches – bringing together training, leadership support, workflow integration, infrastructure, and feedback – rather than single educational or technical interventions alone.^52^ In palliative care, recent systematic reviews continue to highlight ongoing concerns regarding clinical appropriateness, the limits of remote physical assessment, and the need for hybrid models with clear escalation to in-person review when complexity or deterioration emerges, particularly for older or medically unstable patients.^53,54^ This underscores the need for clear clinical governance pathways that define when and how care should pivot from virtual to in-person review, including clear agreed clinical thresholds, escalation responsibility, and documentation within routine care pathways. These findings move beyond describing barriers to highlight clinically meaningful differences in telehealth user profiles that can inform tailored organisational supports, escalation pathways, and real-world service design in palliative care.

These results highlight the influence of culture and attitudes in the workplace and among HCPs, thus emphasising the importance of colleagues and management in encouraging the use of telehealth. Digital health priorities for palliative care research advocate for robust governance systems.^23^ Frameworks, such as the Regional Digital Health Action Plan and the HSE Telehealth Roadmap in Ireland, focus on strengthening digital literacy skills and capacity building, supporting the sustainable delivery of services to palliative care patients in Ireland.^25,51^

In terms of service design, the findings suggest that there are distinct user profiles, ranging from a regular or frequent user to a rare or non-user. These patterns of use need differentiated organisational supports. For example, those who use telehealth rarely may benefit from additional training, reassurance around clinical appropriateness, and clearer guidance on when it is appropriate to use. Whereas those that use it more frequently may want opportunities to share best practices, workflow integration, and protected time. Organisational strategies such as appointing telehealth champions, integrating telehealth into routine care pathways, and providing peer support can help build this supportive culture for differing user profiles, which can help embed telehealth use into routine practice while also accommodating differing user needs. Effective telehealth implementation, therefore, requires coordinated planning amongst key stakeholders and decision-makers that effectively recognises variation in adoption and use.

Governments justify the deployment of telehealth, citing cost reductions, improved self- management, and home-based care delivery as reasons to adopt.^55^ However, findings from our survey indicate that HCPs highlight concerns about the suitability of telehealth for all palliative care patients, particularly when assessing patients’ physical symptoms. Given the potential for rapid clinical deterioration, perhaps a more bespoke service may be required to meet individual needs, reinforcing that “*one size does not fit all.*”^29^

Although telephone consultations are reported as the most common form of communication, this may reflect ease of use, familiarity, confidence, convenience, the type of illness, or lack of resources. HCPs’ use of telephone-based care aligns with the Irish Government’s Sláintecare Strategy, which advocates for more integrated care to support citizens to stay healthy in their homes.^56^ However, the underlying support provided by telehealth care needs to remain adaptable over time, potentially requiring hybrid models that combine remote and in-person care.^57^

Based on these findings, we propose a set of actionable recommendations to enhance the effectiveness and sustainability of telehealth services in palliative care:

1. Tailor telehealth resources for the “moderate-use majority” as most respondents fall into the middle categories (“Rarely,” “Sometimes,” 1–3 h/week), telehealth systems should prioritise activities to promote HCP engagement.
  - Quick, low‑friction access options (e.g. telehealth consultations, messaging).
  - Guided self‑service tools for users who engage intermittently but not intensively.
  - Telehealth platform onboarding design nudges for moderate users to increase their confidence with underused telehealth features.
2. Strengthen support for the “heavy‑use minority.”
  - Support blended schedules and virtual assistance to avoid full days of telehealth sessions or back-to-back virtual blocks without breaks.
  - Provide feedback loop mechanisms where HCPs can report telehealth system pain points and suggest fixes.
  - Recognise and reward quality telehealth engagement through professional development credit.
3. Address those with no or minimal telehealth use.
  - Design tailored digital literacy supports (e.g. how‑to videos, live tech help) and opportunities for protected time to support HCP training.
  - Streamline technical workflows to reduce technical burden by ensuring fast, reliable platform access, minimal login steps, intuitive interfaces, and immediate technical support to promote focus on the task rather than troubleshooting technology.
  - Provide training to support HCPs’ decision-making around the use of telehealth engagements appropriate for diverse palliative care patient populations.

This approach aligns with Nguyen et al., who emphasised the need to balance technology-supported palliative care.^58^ Although it is important to design telehealth that meets the changing requirements of people with palliative care needs, this flexibility in design and subsequent care delivery may have knock-on effects in terms of both direct and indirect costs.

### Limitations

It is important to recognise the limitations of the research. There may be response bias due to self-selection by HCPs choosing to complete the survey. Those with a more positive experience of telehealth could be overrepresented, which affects the external validity of the research, with 63% of respondents indicating prior use. In addition, there is an overrepresentation of females in the survey response; however, this is generally typical in a palliative care workforce. There were missing values, particularly within the outcome variable BI. This is to be expected. The relevant questions asked about the organisation’s expectations and their anticipated use, both of which involve elements of uncertainty that are outside the respondents’ control. The randomness of these missing values was investigated and found insignificant.

It is challenging to get an accurate estimate of the palliative care workforce using publicly available resources in Ireland. The sample size is a small proportion of the overall population of HCPs in palliative care in the Republic of Ireland and Northern Ireland and may not have fully captured the dimensions of the entire population, particularly non-users of telehealth. The sample may overrepresent certain subgroups who are more accessible or willing to participate, thereby skewing the results and potentially affecting the validity of the inferences made. There is a potential risk of Type II errors (false negatives) where the study might not have had sufficient power to detect true effects. A larger sample size would give a more robust representation of intention to use telehealth in palliative care, thereby enhancing the generalisability of our findings. These findings should not be over-generalised but instead be considered as exploratory insights for further investigation.

## Conclusion

While HCPs recognise the benefits of telehealth in palliative care, concerns remain regarding its suitability for managing patients’ physical symptoms. Although the use of telephone-based palliative services is common, there is openness to exploring more hybrid models that combine remote and in-person care. The findings indicate distinct telehealth user profiles, highlighting the need for tailored organisational supports, including targeted training, peer champions, workflow integration, and clear governance pathways for escalation to in-person care within the palliative care workplace. Addressing these factors may enhance HCPs’ adoption and use of telehealth, optimise clinical use, and improve overall patient outcomes and experiences.

## Supplemental Material

sj-doc-1-pcr-10.1177_26323524261437366 – Supplemental material for Healthcare professionals’ experiences of telehealth adoption in palliative care: A cross-sectional survey Supplemental material, sj-doc-1-pcr-10.1177_26323524261437366 for Healthcare professionals’ experiences of telehealth adoption in palliative care: A cross-sectional survey by Ann Kirby, Donal Griffin, Ciara Heavin, Fiona Kiely, Frances J. Drummond and Ciara McGrath in Palliative Care and Social Practice

## Appendix A

**Table A1.** Descriptive statistics of the constructs – full sample of respondents, users, and non-users.

Constructs	Full sample (*n* = 59) *n* (%)	Users (*n* = 37) *n* (%)	Non-users (*n* = 22) *n* (%)
Intention to use			
BI1 My organisation expects me to use telehealth technologies, as part of my job for the delivery of patient care. (*n* = 40 (68% non-response))			
	(*n* = 19)	(*n* = 14)	(*n* = 5)
(Strongly) disagree (score 1–2)	3 (16%)	1 (7%)	2 (40%)
Neutral (score 3)	4 (21%)	3 (21%)	1 (20%)
(Strongly) agree (score 4–5)	12 (63%)	10 (72%)^a^	2 (40%)
BI2 I anticipate using telehealth technologies, where appropriate, in my clinical role in the next 12 months. (*n* = 29 (49% non-response))			
	(*n* = 30)	(*n* = 20)	(*n* = 10)
(Strongly) disagree (score 1–2)	7 (23%)	3 (15%)	4 (40%)
Neutral (score 3)	6 (20%)	2 (10%)	4 (40%)
(Strongly) agree (score 4–5)	17 (27%)	15 (75%)	2 (20%)
BI3 I would like to increase how much I use telehealth technology in my current role. (*n* = 20 (34% non-response))			
	(*n* = 39)	(*n* = 23)	(*n* = 16)
(Strongly) disagree (score 1–2)	11(28%)	9 (39%)	2(13%)
Neutral (score 3)	7(18%)	4 (17%)	3(19%)
(Strongly) agree (score 4–5)	21(54%)	10 (44%)	11(68%)^a^
BI4 I would like to reduce how much I use telehealth technology in my current role. (*n* = 5 (9% non-response))			
	(*n* = 54)	(*n* = 34)	(*n* = 20)
(Strongly)disagree (score 4–5)	28 (52%)	17 (50%)	11 (55%)
Neutral (score 3)	20 (37%)	12 (35%)	8 (40%)
(Strongly) agree (score 1–2)	6 (11%)	5 (15%)	1 (5%)
Performance expectancy			
PE1 I find (or think I would find) telehealth useful in my job			
(Strongly) disagree (score 1–2)	8 (14%)	5 (13%)	3 (14%)
Neutral (score 3)	9 (15%)	5 (14%)^a^	4 (18%)
(Strongly) agree (score 4–5)	42 (71%)	27 (73%)	15 (68%)
PE2 Telehealth enhances (or could enhance) the level of convenience in accessing palliative care services for patients			
(Strongly) disagree (score 1–2)	9 (15%)	6 (16%)	3 (14%)
Neutral (score 3)	7 (12%)	4 (11%)	3 (14%)
(Strongly) agree (score 4–5)	43 (73%)	27 (73%)	16 (73%)
PE3 Telehealth improves (or could improve) the integration of patient care			
(Strongly) disagree (score 1–2)	9 (15%)	7 (19%)	2 (9%)
Neutral (score 3)	12 (21%)^a^	7 (19%)	5 (23%)
(Strongly) agree (score 4–5)	38 (64%)	23 (62%)	15 (68%)
PE4 Telehealth improves (or could improve) access to specialist palliative care for patients			
(Strongly) disagree (score 1–2)	6 (10%)	4 (11%)	2 (9%)
Neutral (score 3)	13 (22%)	7 (19%)	6 (27%)
(Strongly) agree (score 4–5)	40 (68%)	26 (70%)	14 (64%)
PE5 I believe a patient’s physical symptoms may be adequately addressed using telehealth technologies			
(Strongly) disagree (score 1–2)	23 (39%)	14 (38%)	9 (41%)
Neutral (score 3)	21 (36%)	14 (38%)	7 (32%)
(Strongly) agree (score 4–5)	15 (25%)	9 (24%)	6 (27%)
PE6 I believe telehealth technology can support the delivery of good quality care to palliative care patients			
(Strongly) disagree (score 1–2)	12 (20%)	8 (21%)^a^	4 (18%)
Neutral (score 3)	12 (21%)^a^	8 (22%)	4 (18%)
(Strongly) agree (score 4–5)	35 (59%)	21 (57%)	14 (64%)
PE7 I believe telehealth technologies have the potential to support the effective allocation of staff resources			
(Strongly) disagree (score 1–2)	8 (14%)	4 (11%)	4 (18%)
Neutral (score 3)	12 (20%)	7 (19%)	5 (23%)
(Strongly) agree (score 4–5)	39 (66%)	26 (70%)	13 (59%)
PE8 I believe telehealth technologies have the potential to generate cost savings for the health service			
(Strongly) disagree (score 1–2)	4 (7%)	2 (5%)	2 (9%)
Neutral (score 3)	11 (19%)	7 (19%)	4 (18%)
(Strongly) agree (score 4–5)	44 (74%)^a^	28 (76%)	16 (73%)
Effort expectancy			
EE1 I find (or would find) using telehealth technology simple			
(Strongly) disagree (score 1–2)	7 (12%)	3 (8%)	4 (18%)
Neutral (score 3)	17 (29%)	11 (30%)	6 (27%)
(Strongly) agree (score 4–5)	35 (59%)	23 (62%)	12 (55%)
EE2 Learning to use telehealth technologies is (or would) be easy for me			
(Strongly) disagree (score 1–2)	4 (7%)	2 (6%)^a^	2 (9%)
Neutral (score 3)	12 (20%)	9 (24%)	3 (14%)
(Strongly) agree (score 4–5)	43 (73%)	26 (70%)	17 (77%)
EE3 I rarely have (or would expect to rarely have) issues or problems using telehealth technology			
(Strongly) disagree (score 1–2)	18 (30%)	13 (35%)	5 (22%)^a^
Neutral (score 3)	21 (36%)	14 (38%)	7 (32%)
(Strongly) agree (score 4–5)	20 (34%)	10 (27%)	10 (46%)
Facilitating conditions			
FC1 I have access to a reliable internet connection			
(Strongly) disagree (score 1–2)	10 (17%)	5 (14%)	5 (23%)
Neutral (score 3)	6 (10%)	5 (13%)^a^	1 (4%)^a^
(Strongly) agree (score 4–5)	43 (73%)	27 (73%)	16 (73%)
FC2 I have received adequate education and training regarding telehealth technologies			
(Strongly) disagree (score 1–2)	30 (51%)	17 (46%)	13 (59%)
Neutral (score 3)	12 (20%)	7 (19%)	5 (23%)
(Strongly) agree (score 4–5)	17 (29%)	13 (35%)	4 (18%)
FC3 I have access to adequate ICT support in my organisation			
(Strongly) disagree (score 1–2)	15 (25%)	11 (30%)	4 (18%)
Neutral (score 3)	6 (10%)	4 (11%)	2 (9%)
(Strongly) agree (score 4–5)	38 (64%)	22 (59%)^a^	16 (73%)
FC4 I am confident in my organisation’s approach to data security			
(Strongly) disagree (score 1–2)	8 (14%)	5 (14%)	3 (14%)
Neutral (score 3)	6 (10%)	4 (11%)	2 (9%)
(Strongly) agree (score 4–5)	45 (76%)	28 (76%)	17 (77%)
Social influence			
SI1 I am strongly encouraged by my managers to use telehealth technology			
(Strongly) disagree (score 1–2)	14 (24%)	7 (19%)	7 (32%)
Neutral (score 3)	25 (42%)	15 (41%)	10 (46%)
(Strongly) agree (score 4–5)	20 (34%)	15 (40%)^a^	5 (23%)
SI2 I am aware of the routine use of telehealth technologies by colleagues in my organisation			
(Strongly) disagree (score 1–2)	21 (36%)	10 (27%)	11 (50%)
Neutral (score 3)	12 (20%)	7 (19%)	5 (23%)
(Strongly) agree (score 4–5)	26 (44%)	20 (54%)	6 (27%)
SI3 I am aware of colleagues who are enthusiastic about the use of telehealth technology and champion its use in my organisation			
(Strongly) disagree (score 1–2)	21 (36%)	12 (32%)	9 (41%)
Neutral (score 3)	15 (25%)	9 (24%)	6 (27%)
(Strongly) agree (score 4–5)	23 (39%)	16 (43%)	7 (32%)

PE: performance expectancy; EE: effort expectancy; SI: social influence; FC: facilitating conditions; BI: behavioural intention.

BI4 is reverse coded for consistency.

a Adjusted.

**Table A2.** HCP’s characteristics.

Variable	Frequency (%) Full sample, *n* = 59	Frequency (%) Users, *n* = 37 (63%)	Frequency (%) Non-users, *n* = 22 (37%)
Gender			
Male	10 (17%)	6 (16%)	4 (18%)
Female	49 (83%)	31 (84%)	18 (82%)
Age (years)			
25–34	9 (15%)	5 (13%)	4 (18%)
35–44	9 (15%)	7 (19%)	2 (9%)
45–54	25 (42%)	18 (49%)	7 (32%)
55+	16 (28%)	7 (19%)	9 (41%)
Experience (years)			
1–3	10 (17%)	4 (11%)	6 (27%)
4–5	5 (9%)	4 (11%)	1 (5%)
6–10	6 (10%)	4 (11%)	2 (10%)
11+	38 (64%)	25 (67%)	13 (58%)
Role			
Doctor	18 (31%)	11 (30%)	7 (32%)
Nurse	26 (44%)	18 (48%)	8 (36%)
Other practitioners	15 (25%)	8 (22%)	7 (32%)
Location			
Hospice	18 (31%)	8 (22%)	11 (50%)
Hospital	6 (10%)	2 (5%)	4 (18%)
Community	19 (33%)	16 (43%)	3 (14%)
Located in 2+ settings	15 (26%)	11 (30%)	4 (18%)
Management role			
Yes	22 (43%)	14 (45%)	8 (40%)
No	24 (47%)	14 (45%)	10 (50%)
Other	5 (10%)	3 (10%)	2 (10%)
Type of contract			
Full time	37 (63%)	23 (62%)	14(64%)
Part time (>0.5 WTE)	19 (32%)	13 (35%)	6 (27%)
Part time (<0.5 WTE)	3 (5%)	1 (3%)	2 (9%)

WTE: whole time equivalent; HCP: healthcare professional.

**Table A3.** Mean scores for constructs.

Constructs	Mean (SD)	Users (*n* = 37) Mean (SD)	Non-users (*n* = 22) Mean (SD)	*p* Value of users and non-users
BI	3.45 (0.75)	3.52 (0.79)	3.33 (0.67)	0.35
PE	3.60 (0.77)	3.61 (0.74)	3.57 (0.84)	0.10
EE	3.44 (0.64)	3.38 (0.58)	3.53 (0.74)	0.39
FC	3.53 (0.82)	3.53 (0.71)	3.51 (0.99)	0.91
SI	3.08 (0.84)	3.23 (0.90)	2.85 (0.69)	0.10
Little test result		Chi-square distance = 4.50	Degrees of freedom = 9	Prob > chi-square = 0.876

PE: performance expectancy; EE: effort expectancy; SI: social influence; FC: facilitating conditions; BI: behavioural intention.

BI contains missing values therefore was adjusted accordingly see Little test result, Mann Whitney *U* for PE as it is not normally distributed.

**Table A4.** Cronbach alpha results.

Scales	BI	EE	PE	FC	SI
Average inter-item covariance	0.435	0.266	0.54	0.491	0.516
Number of items in scale	4	3	8	4	3
Scale reliability coefficient	0.712	0.642	0.915	0.736	0.729

PE: performance expectancy; EE: effort expectancy; SI: social influence; FC: facilitating conditions; BI: behavioural intention.

BI = BI1–BI3, EE = EE1–EE3, PE = PE1–PE8, FC = FC1–FC4, SI = SI1–SI3.
